# Injury rate and risk factors among small-scale gold miners in Ghana

**DOI:** 10.1186/s12889-019-7560-0

**Published:** 2019-10-24

**Authors:** Emmanuel Kweku Nakua, Ellis Owusu-Dabo, Samuel Newton, Adofo Koranteng, Easmon Otupiri, Peter Donkor, Charles Mock

**Affiliations:** 1 School of Public Health, Department of Epidemiology and Biostatistics, Kwame Nkrumah University of Science and Technology, Kumasi, Ghana; 2 Department of Surgery, Kwame Nkrumah University of Science and Technology, Kumasi, Ghana; 3 Department of Population, Family and Reproductive Health, Kwame Nkrumah University of Science and Technology, Kumasi, Ghana; 4 University of Washington, Seattle, USA

**Keywords:** Small-scale, Mining, Gold, Safety, Personal protective equipment, injury

## Abstract

**Background:**

To determine the potential risk factors for injury, estimate the annual injury rate and examine the safety perceptions, and use of personal protective equipment among small-scale gold miners in Ghana.

**Methods:**

A cross-sectional survey was carried out with 494 small-scale gold miners from four major mining districts in Ghana. A household-based approach was used to obtain a representative sample of miners. The study was conducted from June 2015 to August 2016. A systematic sampling technique was used to select households and recruit respondents to interview. Miners were asked about any mining related injury that they had sustained in the past year. A logistics regression model was employed to examine the association between risk factors and injury. Data were analyzed with STATA version 14.0.

**Results:**

The annual incidence rate of mining-related injury was 289 per 1000 workers. Injuries were mainly caused by machinery/tools 66(46.1%), followed by slip/falls 46(32.2%). The major risk factor for injury was underground work (adjusted odds ratio for injury 3.19; 95% CI = 1.42–7.20) compared with surface work. Higher education levels were protective, with adjusted odds ratios of 0.48 (95% CI = 0.24–0.99) for middle school education and 0.38 (95% CI 0.17–0.83) for secondary school compared with no schooling. Only 15(3.0%) of miners reported to have had safety training in the past year and 105(21.3%) indicated that there were safety regulations at their work place. A moderate number of workers reported using work boots 178(36.0%) and hand gloves 134(27.1%), but less than 10% of workers used other personal protective equipment.

**Conclusion:**

The annual injury incidence rate among small-scale gold miners is high. Potential targets for improving safety include increasing safety training, increasing use of personal protective equipment, and better understanding potential changes that can be made in the machinery and tools used in small-scale mining, which were associated with almost half of all injuries.

## Background

Over the past decade, small-scale mining has undergone massive proliferation in most low- and middle-income countries (LMICs) due to high demand for precious metals and the marketability of gold [1, 2]. Employing over 100 million workers both directly and indirectly, the small-scale mining sector contributes significantly to the development of global economies [3]. Despite the economic contributions of small-scale mining, its operations are seen to cause considerable damage to the environment and to contribute substantially to occupational injuries [4]. It is estimated that in this sector, across over 30 countries, about 13 million of the workers carry out most of their activities under unfriendly, harsh, and risky conditions, and with low pay [5]. This problem is not peculiar to Ghana as it runs across most countries where gold is found, and the magnitude of the problem is mostly experienced in the informal mines or what is locally referred to as “*galamsey*”. Although much small-scale gold mining is illegal in Ghana, its patronage is high, contributing to about a third of all the gold production in the country [6].

Meanwhile, the lack of safety regulations, law enforcement, education, training, functional infrastructure, and equipment are the commonly reported reasons that are believed to have increased injuries among small-scale miners in most LMICs, including Ghana [7]. For instance, a report from the International Labor Organization (ILO) stated that small-scale miners are 6–7 times more likely to experience injuries when compared with large scale miners [8]. While engineering control are the preferred approaches to mitigating safety hazards in the mines, these approaches are often not adopted in the resource-limited settings in which most informal small-scale miners work [8].

Ironically, the less-preferred alternatives to reducing occupational risks, including the use of personal protective equipment (PPE, e.g. hardhats, safety glasses, gloves, work boots), also appears to be uncommon among these miners [9]. This raises questions about why even the least expensive and most common method to safeguarding injuries are rejected in the small-scale mining sector. Separate surveys among small-scale miners in some parts of Ghana, revealed that most of the miners have never used any form of PPE [9]. The level of mechanization in most small-scale mining is very low, activities are carried out with inadequate safety precautions, and the techniques employed by small-scale miners are believed to be obsolete which further exposes the workers to injury and other health hazards [10]. Most equipment used in this venture are manually operated, even though they are known to cause more injuries compared to automated equipment.

Aside from the above few studies, little is known about injury rates or risk factors in small-scale gold mining in Ghana. Therefore, this study was initiated to estimate the injury rate among small-scale (“*galamsey”*) gold miners, to determine the risk factors for injury, and to examine the safety perceptions and PPE use among these workers. By so doing, we hoped to identify priority actions that could be taken to improve safety for workers in this sector.

## Methods

### Study design and setting

A cross-sectional survey of small-scale miners was conducted in four mining districts in four different regions of Ghana. The study was conducted over from June 2015 to August 2016. A community-based approach was used in a representative sample of households in the districts. This was done to avoid the “healthy worker bias”. It was assumed that injured workers may stay off work for some time in order to recover before returning to work, therefore interviewing respondents at the household level improves our chances of meeting respondents of interest in comparison to visiting the mining sites [11]. Detailed description of the methodology is reported elsewhere [12].

### Study population and sampling strategy

Small scale informal miners otherwise known as “*galamsey*” were used in this study. The mining industry in Ghana is usually defined according to quantum of capital investment and number of workers. Mining activities in this category are usually surface mining and small tunnel mining. This type of mining is mostly engaged in by individuals with low or no formal education and the operators of these activities often fail to obtain the requisite documentation and license; thus making it illegal. Nonetheless, small-scale mining accounts for approximately 35% of the gold production in Ghana [6].

The researchers recruited individuals who had more than a year working experience, either currently working or had been laid off their post in the last three months to participate in the study. A total of 494 miners were selected from four districts using a probability sampling technique known as probability proportion to size.

### Data collection

The study used trained research assistant to collect data from participants using smartphone loaded with the structured questionnaire. The outcome of interest for this study was self-reported injury in the last 12 month which was related to any mining activities prior to the interview date. Persons with this outcome that cause at least a day loss of activity for which medical care was sought or not in the year preceding the study were interviewed. The questionnaire for the study was self-designed and face-to-face interviewer-administered. The questionnaire was structured in English language; however, it was translated into local language (Twi) during the interview in some instances. The interviewers were trained on how to translate the questionnaire into Twi and back to English in order to maintain their meaning. Data collected from the participants included demographic characteristics, nature of the mining work of participant and risk factors. Questions on training availability, safety procedures and rules availability for workers protection were asked. The most recent injury information was recorded from participants, with data on multiple injury episodes not collected.

### Ethical consideration

The study was approved by the Kwame Nkrumah University of Science and Technology (KNUST)/Komfo Anokye Teaching Hospital (KATH) Committee on Human Research, Publications and Ethics (CHRPE) in Ghana. Written informed consent was obtained, after a trained interviewer explained the purpose of the study to the household head and then selected members of the household to interview. Confidentiality of their identity was assured and respondents were free to withdraw from the study or refuse to answer any questions they so deemed. Names or other identifying information were not collected.

### Data management

Data were collected using electronically using smartphones with the aid of open data kit (ODK) software loaded with the questionnaire template. Collected data was uploaded to a secured google cloud server online. The data was later downloaded in excel format and imported in Stata 14.0 statistical software, cleaned and for analysis. Data completeness and consistency were checked by using a self-written program in Stata.

### Statistical analysis

Descriptive analysis used frequencies and percentages for categorical variables to summarize data. Relationship of categorical variables was assessed using the Chi Square test and Fisher exact test where expected values were less than 5. The annual injury incidence rate was estimated based on the number of injuries divided by the total number of small-scale workers interviewed. As recall period was one year, the rate derived was an annual rate [13].

Univariate and multivariable logistic regression models were used to predict factors influencing odds of injury at the work place. The multivariable logistic regression model incorporated variables that were independently significant in the univariate analysis (*p* ≤ 0.05) as well as variables that were considered to be important or potentially confounding. Main exposure variables considered a priori were department of work, years of experience at the mines and tobacco use. The non-significant but important characteristics were controlled in the backward regression model. The fit of the model was assessed using the log maximum likelihood test. Significance level of 0.05 considered with a 95% confidence interval. The statistical analysis of the household survey was conducted using the STATA version 14.0 (Statacorp, College Station, USA) statistical software package.

## Results

The 494 small-scale miners who participated in this study were primarily males (95.1%), youthful (42.7% between 18 and 25 years), and single (54.7%). They were literate, with only 9.7% never been to school. About 71.9% had five or less years of mining experience. They were mostly permanent employees (39.9%) and casual labourers (40.5%). A little over half (53.6%) of the mining activities were surface, while open pit mining constituted 38.3%. Univariate analysis, showed that age group and department of work were significantly associated with risk of injury, see Table 1.

**Table 1:** Socio demographic characteristics of study sample

Characteristics		Injuries			
		No *n* = 351(%)	Yes *n* = 143(%)	Total *n* = 494 (%)	*P*-value
Age group					0.003
18–25		160 (45.6)	51 (35.9)	211 (42.7)	
26–35		112 (31.9)	65 (45.8)	177 (35.8)	
36–45		62 (17.7)	14 (9.9)	76 (15.4)	
≥ 46		17 (4.8)	12 (8.5)	29 (5.9)	
Missing			1	1	
Sex					0.37
Female		19 (5.4)	5 (3.5)	24 (4.9)	
Male		332 (94.6)	138 (96.5)	470 (95.1)	
Marital Status					0.46
Single		196 (56.2)	74 (52.5)	270	
Married		153 (43.8)	67 (47.5)	220	
Missing		2	2	4	
Education level					0.07
Non-Educated		27 (7.7)	21 (14.7)	48 (9.7)	
Primary		65 (18.5)	32 (22.4)	97 (19.6)	
JSS/Middle		165 (47.0)	60 (42.0)	225 (45.6)	
SSS		89 (25.4)	27 (18.9)	116 (23.5)	
Tertiary		5 (1.4)	3 (2.1)	8 (1.6)	
Mining experience (Years)					0.39
1–5		254 (74.7)	101 (72.1)	355 (74.0)	
6–10		68 (20.0)	26 (18.6)	94 (19.6)	
10–20		14 (4.1)	11 (7.9)	25 (5.2)	
21+		4 (1.2)	2 (1.4)	6 (1.3)	
Missing		11	3	14	
Work status					0.61
Employee		135 (39.6)	62 (44.3)	197 (41.0)	
On-contract		60 (17.6)	24 (17.1)	84 (17.5)	
Casual		146 (42.9)	54 (38.6)	200 (41.6)	
Missing		10	3	13	
Work Department					0.001
Underground Mine		15 (4.4)	19 (13.3)	34 (7.0)	
Surface Mine		201 (58.3)	64 (44.8)	265 (54.3)	
Open Pit		129 (37.4)	60 (42.0)	189 (38.7)	
Missing		6		6	

There were a total of 143 mining-related injuries reported, for an annual incidence rate of 289 injuries per 1000 workers. Rates did not differ significantly among different categories of work status: employee (314 injuries per 1000 workers); on-contract (286) and casual (270). The number of days lost from work was used as a measure of the severity of the injury sustained [8]. Of the 143 injured workers, 32.9% of them were deemed to have had minor injuries (“no day lost” up to six days of absence from work), 30.0% were moderate (7–29 days of absence) and 37.1% were severe (absence for more than 30 days). The two most common types of injuries were contusion/abrasion (45.5%) and laceration (39.8%), with a smaller proportion (14.7%) being fractures sustained in various body parts.

Injuries reported were as a result of miners’ use of machinery or tools (46.1%), followed by slip and falls (32.2%), with less than 2 % resulting from burns. Meanwhile, over 80 % (81.8%) of these injured workers had returned to work as at the time of this survey (see Table 2).

**Table 2:** Percent distribution of type of injury, causes of injury and severity of injury

Variables	Frequency	Percent (%)
Production target		
Yes	178	36.0
No	308	62.4
Missing	8	1.6
Type of Injury		
Contusion/abrasion	65	45.5
Fracture	21	14.7
Laceration	57	39.8
Mechanism of Injury		
Fall from height	11	7.7
Struck/hit by falling object	18	12.6
Machinery/tool	66	46.1
Slip/Falls	46	32.2
Burns	2	1.4
Body part injured		
Head	20	14.0
Neck	5	3.5
Back	8	5.5
Chest	5	3.5
Legs	59	41.3
Arms	35	24.5
Others	11	7.7
Medical Attention		
No medical care	41	28.7
Hospitalized	56	39.2
Treated and discharge same day	46	32.1
Severity of Injury^b^		
Minor	47	32.9
Moderate	43	30.0
Severe	53	37.1
Returned to Work		
Yes	117	81.8
No	26	18.2

The use of various forms of personal protective equipment (PPE) among mineworkers is summarized in Table 3. The attitude of workers on use of PPE is poor if training and enforcement at the workplace are not emphasized, which is the case among small-scale mine workers. The commonest PPE used were hand gloves (27.1%) and work boots (36.0%). The ear protectors were the least utilized PPE (2.0%).

**Table 3:** Personal protective equipment use among miners

Type of personal protective equipment	Frequency	Percent (%)
Thermal protective coat (flame retardant coat)	7	1.4
Thermal protective pants (flame retardant coat)	4	0.8
Hand gloves	134	27.1
Helmet	26	5.3
Hood	7	1.4
Eye protection (safety glasses)	21	4.3
Eye protection (face shield)	11	2.2
Work boots (steel toe, heat resistant, chemical resistant)	178	36.0
Reflective vest	6	1.2
Ear protectors (ear muff and ear plug)	10	2.0

Table 4 shows the analysis of safety perception against risk of injury. The majority of respondents did not know of any safety regulations at their worksite. However, of those who knew of safety regulations, the majority did observe them. Job satisfaction was low and few workers had ever had safety training. Nonetheless, none of these factors was associated with risk of injury.

**Table 4:** Safety perceptions of injured against non-injured persons

Characteristics		Non-injured	Injured	P-value
Rules guiding the safety of individuals at work	No	284 (80.9%)	105 (73.4%)	0.07
	Yes	67 (19.1%)	38 (26.6%)	
Do you often comply with these regulations during work?	Always	52 (77.6%)	28 (73.7%)	0.45
	When necessary	7 (10.5)	7 (18.4%)	
	Not Sure	8 (11.9%)	3 (7.9%)	
Job satisfaction	No	295 (84.1)	114 (79.7%)	0.25
	Yes	56 (16.0%)	29 (20.3%)	
Attendance of workshop on mining safety in the past 12 months	No	340 (96.9%)	139 (97.2%)	0.55
	Yes	11 (3.1%)	4 (2.8%)	

The effect of several risk factors has already been detailed. These and other risk factors are summarized in Table 5. In the univariate analysis, age group, education, department of work, and tobacco use are associated with risk of injury. From multivariable analysis, the major risk factor for injury was underground work (adjusted odds ratio 3.19; 95% CI = 1.42–7.20) compared with surface work. Higher education levels were protective, with adjusted odds ratios of 0.48 (95% CI = 0.24–0.99) for middle school education and 0.38 (95% CI 0.17–0.83) for secondary school compared with no schooling.

**Table 5:** Factors influencing injury in the mines

Variables	Crude OR (95% CI)	*P*-Value	AOR (95% CI)	*P*-value
Age group				
18–25	1.00		1.00	
26–35	1.82 (1.17, 2.82)	0.007	1.57 (0.95, 2.61)	0.08
36–45	0.71 (0.37, 1.37)	0.31	0.54 (0.25, 1.17)	0.12
46+	2.21 (0.99, 4.94)	0.05	1.37 (0.53, 3.52)	0.51
Sex				
Female	1.00			
Male	1.58 (0.58, 4.31)	0.37		
Marital Status				
Single	1.00			
Married	1.16 (0.78, 1.72)	0.46		
Education level				
Non-Educated	1.00		1.00	
Primary	0.63 (0.31, 1.29)	0.21	0.72 (0.32, 1.61)	0.43
JSS/Middle	0.47 (0.25, 0.89)	0.02	0.48 (0.24, 0.99)	0.05
SHS	0.39 (0.19, 0.80)	0.01	0.38 (0.17, 0.83)	0.02
Tertiary	0.77 (0.17, 3.60)	0.74	0.62 (0.11, 3.61)	0.60
Mining experience (Years)				
1–5	1.00		1.00	
6–10	0.96 (0.58, 1.60)	0.88	0.87 (0.49, 1.57)	0.65
11–20	1.97 (0.87, 4.50)	0.11	2.07 (0.80, 5.31)	0.13
21+	1.26 (0.23, 6.97)	0.79	0.74 (0.09, 6.28)	0.78
Department of work				
Surface Mine	1.00		1.00	
Underground Mine	3.98 (1.91, 8.28)	0.001	3.19 (1.42, 7.20)	0.005
Open Pit	1.46 (0.96, 2.21)	0.07	1.28 (0.79, 2.06)	0.31
Daily Production targets per shift				
No	1.00			
Yes	0.92 (0.61, 1.38)	0.68		
Alcohol Consumption				
No	1.00			
Yes	1.21 (0.82, 1.80)	0.33		
Tobacco Use				
Never Smoked	1.00		1.00	
Ever smoked	1.88 (1.17, 3.03)	0.009	1.34 (0.62, 2.90)	0.46
Currently smokes	1.56 (0.76, 3.19)	0.23	1.61 (0.93, 2.80)	0.09
Days off per month				
0–2 days	1.00			
3–4 days	0.87 (0.56, 1.36)	0.54		
5–8 days	0.64 (0.38, 1.06)	0.08		

OR: Odds ratio. AOR: Adjusted odds ratio

## Discussion

There is growing attention globally to small-scale mining and the associated occupational health risks faced by its workforce. Anecdotal evidence suggest that small-scale mining poses more occupational hazards than what may be found in more regulated or large scale mining operations [14]. This study sought to estimate the incidence of injuries among small-scale gold miners in Ghana and to assess their risk perception and risk factors associated with injury. The study found a very low level of workplace safety rules, very low levels of safety training, moderate use of boots and gloves but low use of other personal protective equipment, and a high rate of injuries. The major risk factors for injury were below ground work and lower education levels.

Findings in this study indicates machinery/tools (equipment) (46.1%) as the main causes of injuries. Slip/falls and fall from height also contributes to injuries among this category of miners. These were consistent with findings by Chimamise et al. (2013) who also reported that equipment’s is the leading cause of injuries in the mines in Zimbabwe. Similarly, an analysis of 5-year mining data revealed an injury rate of 37 to 88% per year of injuries were associated with tools and equipment’s [15]. Furthermore, according to Jenning, (2003) small-scale miners who acquire equipment’s for mining are poorly maintained as a result causes accidents leading to injuries [16]. The tools or equipment used by small-scale miners are designed with inadequate safety precautions, and mostly manually operated. Therefore, workers who use this manual equipment were more likely to sustain severe injuries than those who use automatic equipment. However, on the contrary a study by Michelo, (2009), indicated that fall of rocks as the commonest cause of fatalities [17].

The dangerous nature of the mining environment makes it very important for workers to be alert and to be compliant with safety standards. Small-scale miners who work underground face the danger of pit collapse and slippery grounds. They do not have the monetary capacity to erect concrete walls or pillars to prevent rocks from falling or pits from collapsing, rather they use logs or bamboo sticks to block falling rocks. In the current study the odds of sustaining injury was 3.19 times higher for underground work compared with surface mining.

Production targets have been found to be associated with increased risk of injury in other studies. In the gold mining industry, there is overemphasis on production targets. The desire to achieve targets set make individuals disregard safety rules [18]. In order for employees to achieve targets, they adopt shortcuts and thus jeopardize safety. Surprisingly, the findings of the current study did not indicate any statistical difference between the injured and non-injured workers with regards to production targets (Tables 2 and 5) or awareness of rules and regulation operating within their company guiding the safety of individuals during work (Table 4).

Safety perceptions of miners aid in the observation of safety regulations. There was no significant difference in terms of compliance of safety regulations during work, job satisfaction and seminar or workshop attendance with injury risk in the current study. However, Paul and Maiti, (2007) revealed that among underground coal miners in India, job dissatisfaction has a strong influence on occupational injury. Individuals who are dissatisfied are unable to concentrate on their tasks and as a result, they are unable to observe proper safety practices [19].

The low of workshop or lectures/seminars on mining safety attendance is a worrying situation for the sector (Table 4). New employees usually need some training to master the safety procedures and identify eminent dangers. In South Africa, small-scale miners receive technical skills as well as institutional support to be able to work safely in the mines [20]. Learning on the job, especially in gold mining environment, is very dangerous.

The level of mechanization in small-scale mining is very low and the equipment used lacks adequate safety features. The techniques employed by small-scale miners are also obsolete and expose workers to injury and health hazards [10]. The equipment used by small-scale miners is primarily manually operated. According to a previous study by Chimamise et al., (2010) manually operated equipment causes more injuries than automated equipment. Future directions for research on safety promotion for small scale mining might address increasing the use of the safer automated equipment or potential modifications to manually operated equipment to make it safer, the latter of which has scarcely been addressed in the literature.

The risk from the manual equipment is compounded by lack of PPE use. The low reported PPE use in our study is consistent with findings of other studies of small-scale miners in Ghana and Sub-Sahara Africa. Moreover, the injuries to the legs/feet and head could have been prevented or minimized by higher use of helmets and steel-toed boots. Workers who are engaged in mining ideally should have at least basic education, as well as short exposure to safety training. A miniscule proportion (3.0%) of miners in the current study had any safety training in the past year.

In the multivariable analysis, after adjusting for the effect of other covariates, working underground had an increased risk of injury while education level was a protective factor for injuries. This is consistent with other occupational researchers that have established high risk of injuries among mineworkers with no formal education. Often those engaged in small-scale mining are not well educated which makes them less prepared to understand safety procedures and thus more likely to commit human errors [21].

The finding of increased risk of injury with tobacco use seems incongruous. This may be because marijuana is often mixed with tobacco locally. Hence, this variable may reflect the effect of substance-abuse more broadly, rather than tobacco use alone.

Our study has a number of limitations. First, the rate of injury is likely under-estimated due to the cross-sectional and retrospective nature of the study leading to recall bias, which would tend to selectively capture the more severe injuries. Second, although we did use a household survey to overcome the healthy-worker bias, some of the more severely injured miners might have left the mining areas to go back to their families for support and care, leading to further under-counting, thus incompletely addressing the healthy worker bias. Third, the enrollment criteria included those currently working and those who had been laid off in the past 3 months. Data were not collected as to which criteria a given participant met. Hence, we were not able to calculate rates separately for each group. Despite these limitations, this study has several strengths, including the fact that it involved a large sample size of randomly selected workers across four geographically separate mining areas. Given that there are no lists of these informal workers, the use of random household selection makes the sample as generalizable as is possible under the current circumstances.

## Conclusions

The annual injury incidence rate among small-scale gold miners in Ghana was high. Many of the injuries were severe, with more than a third resulting in a month or more of disability. The leading mechanism of injury was use of machinery/tools. Safety training was almost non-existent and use of personal protective equipment was low. Potential targets for improving safety include increasing safety training, increasing use of personal protective equipment, and better understanding potential changes that can be made in the machinery and tools used in small-scale mining.

## Abbreviation

CHRPE: Committee on Human Research Publications and Ethics
ILO: International Labor Organization
KATH: Komfo Anokye Teaching Hospital
KNUST: Kwame Nkrumah University of Science and Technology
LMICs: Low- and Middle-Income Countries
PPE: Personal Protective Equipment

## References

[1] Organization, W.H (2016). Environmental and occupational health hazards associated with artisanal and small-scale gold mining.

[2] Council, A.G. Reducing mercury use in artisanal and small-scale gold mining: a practical guide; final technical report-output: United Nations Environment Programme; 2012.

[3] Gavin H, Clive P (2003). Why is illegal gold mining activity so ubiquitous in rural Ghana?. Afr Dev Rev.

[4] Aryee NAB, Ntiberry KB, Atorkui E (2002). Trends in the small-scale mining of precious minerals in Ghana: a perspective on its environmental impact. J Clean Prod.

[5] Gyekye SA (2003). Causal attributions of Ghanaian industrial workers for accident occurrence: miners and non-miners perspective. J Saf Res.

[6] McQuilken, J. and G. Hilson, Artisanal and small-scale gold mining in Ghana. Evidence to inform an 'action dialogue*'. http://pubs.iied.org/16618*IIED. 2016: IIED, London.

[7] Wilson M (2015). Integrated assessment of artisanal and small-scale gold mining in Ghana—part 3: social sciences and economics. Int J Environ Res Public Health.

[8] International Labor Organization (ILO) (1999). Social and labour issues in small-scale mines. Report for discussion at the tripartite meeting on social and labour issues in small-scale mines.

[9] Chimamise C (2013). Factors associated with severe occupational injuries at mining company in Zimbabwe, 2010: a cross-sectional study. Pan Afr Med J.

[10] Teschner BA (2012). Small-scale mining in Ghana: the government and the galamsey. Resour Policy.

[11] Li C-Y, Sung F-C (1999). A review of the healthy worker effect in occupational epidemiology. Occup Med.

[12] Nakua EK, et al. Occupational injury burden among gold miners in Ghana. Int J Inj Control Saf Promot. 2019:1–7. 10.1080/17457300.2018.1515232 31164051

[13] McGee K (2004). Guidelines for conducting community surveys on injuries and violence. Inj Control Saf Promot.

[14] Calys-Tagoe BN (2017). A comparison of licensed and un-licensed artisanal and small-scale gold miners (ASGM) in terms of socio-demographics, work profiles, and injury rates. BMC Public Health.

[15] Kecojevic V (2007). An anlaysis of equipmemt-related fatal accidents in U.S. mining operations: 1995-2005. Saf Sci.

[16] Jennings N. *Addressing labour and social issues in small-scale mining*, in *The Socio-Economic Impacts of Artisanal and Small-Scale Mining in Developing Countries*: AA Balkema The Netherlands; 2003. p. 151–60.

[17] Michelo P, Bratveit M, Moen BE (2009). Occupational injuries and fatalities in copper mining in Zambia. Occup Med (Lond).

[18] Probst TM, Brubaker TL (2001). The effects of job insecurity on employee safety outcomes: cross-sectional and longitudinal explorations. J Occup Health Psychol.

[19] Paul P, Maiti J (2007). The role of behavioral factors on safety management in underground mines. Saf Sci.

[20] Ledwaba P (2017). The status of artisanal and small-scale mining sector in South Africa: tracking progress. J South Afr Inst Min Metall.

[21] Kunar B, Bhattacherjee A, Chau N (2010). A matched case – control study of occupational injury in underground coal mine workers. The journal of the Southern African Institute of Mining and Metallurgy.

